# Protective Role of Folic Acid and Vitamin B12 Against Chronic Ivermectin‐Induced Reproductive Damage: Oxidative Stress, Immunohistochemical Changes (Caspase‐3, Bax, BCL2, and PCNA Expressions) and Mitochondrial 16S rRNA Analysis

**DOI:** 10.1002/cbdv.71861

**Published:** 2026-10-22

**Authors:** Yahia A. Amin, Asmaa M. Mosa, Rana A. Ali, Seham A. Mobarak, Zeinab A. Mar'ie

**Affiliations:** ^1^ Department of Theriogenology Faculty of Veterinary Medicine Aswan University Aswan Egypt; ^2^ Department of Zoology Faculty of Science Qena University Qena Egypt; ^3^ Department of Zoology Faculty of Science Hurghada University Hurghada Egypt

**Keywords:** folic acid, genetic variation, immunohistochemical changes, ivermectin, oxidant/ antioxidant profile, vitamin B12

## Abstract

This study aims to investigate the protective effects of folic acid (FA) and vitamin B12 (Vit.B12) against high‐dose effects of IVM on reproductive hormones, semen quality, oxidant/antioxidant profile in testicular tissue, histopathological changes, and immunohistochemical investigation (Caspase‐3, Bax, BCL2, and PCNA) in the testes of male rats. Twenty eight rats were divided into four equal groups: Control, Ivermectin, Ivermectin + Folic acid, and Ivermectin + Vit.B12. IVM was administered at 5 mg/kg/day, folic acid at 20 mg/kg, and vitamin B12 at 50 µg, with the respective routes and 30‐day treatment period specified. Testosterone and semen parameters were evaluated. Testicular specimens were examined for oxidant/antioxidant markers, histological changes, and immunohistochemical expressions. Mitochondrial 16S‐rRNA sequences were analyzed and deposited in GenBank under accession numbers PP320440.1–PP320443.1. Results showed that IVM significantly reduced testosterone level, sperm count, and antioxidants, while increasing oxidative stress. Seminiferous tubules damage was observed in the IVM group. Immunohistochemical analysis showed strong caspase‐3 and Bax expressions in the IVM group. BCL2 and PCNA showed altered expression patterns. 16S‐rRNA sequence analysis revealed limited sequence variation among the experimental groups. Co‐treatment with folic acid or Vit.B12 significantly improved all parameters affected by IVM. These findings indicate that FA and Vit. B12 may provide protective effects against IVM‐associated testicular alterations.

## Graphical abstract

Twenty eight rats were divided into four groups (7 rats per group): Control group, Ivermectin group (IVM group), Ivermectin + Folic acid group (IVM + FA group), and Ivermectin + Vit.B12 group (IVM + Vit.B12 group). Blood and semen samples were collected to assess testosterone and semen parameters. Testicular specimens were examined for oxidant/antioxidant markers, histological changes, and immunohistochemical expressions (caspase‐3, Bax, BCL2, and PCNA). Mitochondrial 16S rRNA sequences were also analyzed and deposited in GenBank under accession numbers PP320440.1–PP320443.1.

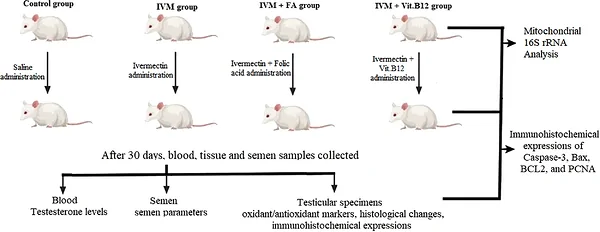

## 1 Introduction

Ivermectin (IVM) is a widely used antiparasitic drug in both animals and humans [1]. Beyond its antiparasitic activity, IVM has been shown to possess antiviral [2], antibacterial [3], and anticancer [4] properties. Although generally considered safe at therapeutic doses [5], repeated or high‐dose exposure to IVM has been associated with adverse reproductive effects. Specifically, it can lead to reduced testicular volume, decreased tubular diameter, and thinning of the germinal epithelium [6]. IVM has been shown to impair sperm production and induce morphological abnormalities in sperm due to damage in the testicular tissue [7]. Previous studies found that weekly administration of IVM for 8 weeks resulted in significant fertility disturbances [8].

Histological analysis has revealed that IVM causes nuclear alterations and clustering of cellular organelles in germ cells, which interfere with normal meiotic divisions and affect sperm morphology [7]. These disruptions—manifested as impairments in spermatogenesis and spermiogenesis—are attributed to disorganization of cells across different stages of the seminiferous epithelium cycle [6]. Additionally, high doses of IVM have been associated with reduced testosterone levels [6, 9]. As a result, IVM may negatively impact animal reproduction, potentially reducing livestock productivity such as milk and meat yield‐posing a broader concern for food security. Therefore, identifying agents that can mitigate the reproductive toxicity of this widely used drug is of critical importance.

Substances with protective effects against reproductive toxicity are diverse, with B vitamins‐particularly folic acid (vitamin B9) and vitamin B12‐being among the most notable. The properties of B vitamins make them attractive candidates for use as monotherapy or as adjuncts in combination with other drugs, especially due to their anti‐inflammatory and neuroprotective effects. These vitamins have been implicated in the management of various conditions, including rheumatologic diseases, degenerative spinal disorders, polyneuropathies (particularly diabetic neuropathy), and different phases of postoperative recovery [10].

Increasing evidence suggests that oxidative stress and apoptosis may contribute to ivermectin‐induced testicular injury. Excessive reactive oxygen species (ROS) can disrupt cellular redox homeostasis, promote oxidative damage, and trigger mitochondrial‐dependent apoptotic signaling. In this pathway, oxidative imbalance may favor the activation of proapoptotic BCL2 family proteins such as Bax, mitochondrial membrane permeabilization, cytochrome c release, and subsequent activation of caspase‐3, whereas BCL2 contributes to cellular survival by opposing these apoptotic events [11]. In male rats, ivermectin exposure has been associated with testicular oxidative stress, pathological alterations, and apoptotic changes, including increased caspase‐3 expression and DNA damage [7, 11, 12]. These observations provide a mechanistic rationale for evaluating oxidative‐stress markers together with apoptosis‐ and proliferation‐related markers in the present study. Accordingly, MDA, SOD, CAT, and TAC were assessed to characterize the oxidant/antioxidant balance, while Caspase‐3, Bax, BCL2, and PCNA were examined to evaluate apoptotic and cellular proliferative responses.

Folic acid (FA), the synthetic form of folate, is commonly found in fortified foods and dietary supplements [12]. It is a water‐soluble B vitamin (B9) [13], essential for optimal cellular function and growth. FA plays a crucial role in DNA synthesis, amine metabolism, amino acid synthesis, and cell division [14]. In the context of reproductive health, folic acid has been shown to support spermatogenesis and improve sperm morphology and concentration, primarily due to its antioxidant properties [15]. Furthermore, recent studies have demonstrated that in cases of acetamiprid‐induced reproductive toxicity, rats treated with folic acid exhibited significantly improved reproductive parameters compared to those exposed to acetamiprid alone [16].

Vitamin B12 is a water‐soluble compound essential for the formation of blood cells and DNA [17]. It plays a critical role in cellular metabolism, particularly in the metabolism of amino acids and fatty acids, as well as in DNA synthesis. Additionally, vitamin B12 contributes to maintaining DNA integrity, a factor that has been linked in improvements of semen quality, including enhanced sperm concentration and motility [18]. Recent researches confirmed that Vit.B12 showed protective effects against ivermectin‐induced toxicity in some organs such as liver and kidney [19, 20].

The current study hypothesizes that the administration of folic acid (FA) and vitamin B12 can exert protective effects and mitigate the toxic impact of high‐dose ivermectin on reproductive function and testicular tissue. Specifically, the study aims to evaluate the ameliorative potential of FA and vitamin B12 against the adverse effects of ivermectin exposure on genetic variation, reproductive hormone levels, semen quality, oxidative/antioxidative balance, histopathological changes, and immunohistochemical markers. These markers include the expression of caspase‐3, Bax, BCL2, and proliferating cell nuclear antigen (PCNA) in male albino rats.

## 2 Materials and Methods

### 2.1 Ethics Approval and Drugs

All procedures were carried out according to the guide that was approved by the Ethics Committee of the Faculty of Science, South Valley University, Egypt (approval number 005/06/24). All methods were performed in accordance with the relevant guidelines and regulations. All animal procedures complied with the ARRIVE guidelines

IVM (Bomectin injection) produced by Bayer company (Germany). Folic acid (FA) tablets, produced by El‐Nile Company for pharmaceuticals and chemical industries, dissolved in adequate amount of distilled water. Vit.B12 (Depovit B12), produced by Amriya for pharmaceutical industries‐Egypt, Vit.B12 ampoule dissolved in distilled water to facilitate the obtained dose.

### 2.2 Experimental Protocol

Twenty‐eight adults’ male Wistar albino rats aged between 13 and 15 weeks and weighing an average of 200 ± 20 g were obtained from the Laboratory Animal Housing Unit of the Faculty of Veterinary Medicine at Qena University in Egypt. The animals were housed in plastic cages and maintained under standard laboratory conditions with free access to a commercial standard diet and water ad libitum. Prior to the experiment, all rats were acclimatized for 2 weeks. Graphical abstract illustrated the protocol of the study.

The rats were randomly divided into four groups (*n* = 7 per group) as follows:

- Group 1 (Control Group): Received a saline solution (0.9% NaCl) orally.
- Group 2 (IVM Group): Administered ivermectin (IVM) at a dose of 5 mg/kg/day by subcutaneous injection [21].
- Group 3 (IVM + FA Group): Coadministered IVM (same dose and route as Group 2) and folic acid (FA) at a dose of 20 mg/kg via oral route in distilled water [22].
- Group 4 (IVM + Vitamin B12 Group): Coadministered IVM (same dose and route as Group 2) and vitamin B12 at a dose of 50 µg via intramuscular injection [23].

The duration of the experimental treatment was 30 days.

Graphical abstract illustrated the protocol of the study

Ctrl group: Control group; IVM group: Ivermectin group; IVM + FA group: Ivermectin + Folic acid group; IVM + Vit. B12 group: Ivermectin + Vitamin B12 group

### 2.3 Euthanasia Procedure

At the end of the experimental period, all animals were humanely euthanized for the purpose of sample collection. Rats were individually placed in an induction chamber and anesthetized by inhalation of 3% isoflurane delivered in 100% oxygen at a flow rate of 1–2 L/min. The animals were monitored closely for signs of deep anesthesia, including the absence of pedal withdrawal and corneal reflexes, to ensure full unconsciousness. Once anesthesia was confirmed, euthanasia was carried out through decapitation. In cases where rapid tissue harvesting was critical, decapitation was performed immediately following deep anesthesia to ensure the animals did not regain consciousness. All procedures were performed by trained personnel and were in compliance with institutional and national ethical guidelines for animal experimentation. After euthanasia, relevant tissues and organs were rapidly harvested [24].

### 2.4 Blood Collection and Testosterone Determination

At the end of the 30‐day experimental period, all animals were anesthetized, and blood samples were collected via the retro‐orbital sinus. Following blood collection, the animals were humanely euthanized, and the relevant tissues and organs were rapidly harvested. The blood was collected into a blank tube and was left to coagulate and centrifuged at 3000 rpm for 15 min to separate serum for determination of testosterone. Testosterone determination was performed using Elecsys Testosterone II (Roche Diagnostics).

### 2.5 Semen Analysis

For semen analysis, a small cut like the scissor shape was made in the epical part of the cauda, putting the cauda epididymis in a Petri dish containing 0.5 mL saline and then sperms from the petri dish are examined for their motility within 1min at maximum. For sperm counting, dilution solution was made by adding 900 micron of distilled water into 100 micron of the saline contain sperms, and the hemocytometer was used in sperms counting [25].

### 2.6 Preparation of Tissue Homogenate and Oxidative Analysis

A portion of the testicular tissue was manually homogenized. Tissue homogenates were prepared using 1.0 mL of phosphate buffer for every 100 mg of tissue. The homogenates were then centrifuged at maximum speed at 4°C, and the resulting supernatants were collected for biochemical analyses. Malondialdehyde (MDA) levels were measured using Rat Malondialdehyde, MDA ELISA Kit with SKU CODE: RTEB1739. Superoxide dismutase (SOD) activity was assessed based on Rat Superoxide Dimutase (SOD) ELISA Kit with Catalog Number: MBS036924. Catalase (CAT) activity was determined Rat Catalase (CAT) ELISA Kit with Catalog Number: MBS726781. Total antioxidant capacity (TAC) was measured using Rat Total Antioxidant Status ELISA Kit with Catalog Number: MBS1600693.

### 2.7 Tissue Processing, Sectioning, and Staining

At the end of the experimental period, the rats were euthanized, and both the left and right testes were excised, weighed, and processed for histological examination. The testes were fixed in 10% neutral buffered formalin, followed by a graded alcohol dehydration series (two changes each of 70%, 90%, and 100% ethanol). The tissues were then cleared in xylene, embedded in molten paraffin, and allowed to cool to form solid tissue blocks suitable for sectioning.

Histological sections, 5 µm thick, were obtained from the paraffin‐embedded testicular tissue blocks and stained using Hematoxylin and Eosin (H&E). The staining procedure involved the following steps: deparaffinization in xylene, rehydration through a descending alcohol series (100%, 90%, and 70% ethanol), and rinsing in distilled water. The sections were then stained with hematoxylin, washed under running tap water, and differentiated in 1% acid alcohol (1% HCl in 70% ethanol). Following this, the slides were blued in tap water, rinsed in distilled water, counterstained with eosin, and again rinsed in distilled water. Finally, the sections were dehydrated through an ascending alcohol series (70%, 90%, and 100% ethanol), cleared in xylene, and mounted using Distrene Polystyrene Xylene (DPX) mounting medium [26].

### 2.8 Immunohistochemical Investigation

Expression of the apoptotic proteins BCL2‐associated X (Bax) [27], B cell lymphoma 2 (BCL2) [27], caspase‐3 [28], and proliferating nuclear antigen (PCNA) [29] in the testis tissues was determined immunohistochemically. Paraffin‐embedded tissue sections of 3 µm thickness were rehydrated first in xylene and then in graded ethanol solutions, the slides were then blocked with 5% bovine serum albumin (BSA) in tris‐buffered saline (TBS) for 2 h. After that, the sections were immunostained with the primary antibody: Bax (catalog number: PA5‐ 11378), BCL2 (catalog number: PA5‐20068), or caspase 3 (catalog number: PA1‐29157) (Thermo Fisher Scientific, USA) and PCNA (catalog number: DS‐0360‐E from Diagnostic biosystem, USA) at a concentration of 1 µg/mL containing 5% BSA in TBS and incubated overnight at 4°C. After washing the slides with TBS, the sections were incubated with the corresponding goat antirabbit secondary antibody. Washing with TBS and incubation for 10 min in 0.02% diaminobenzidine containing 0.01% hydrogen peroxide were then performed. Counter staining was carried out using hematoxylin, and the slides were visualized using a light microscope [30]. The quantification of immunostaining was performed using the Image‐J image analysis software (NIH, USA) and presented as the optical density of the stained sections per field (× 100).

For quantitative evaluation of immunohistochemical staining, digital images of the testis sections were analyzed using ImageJ software. The *Integrated Density (IntDen)* value was measured for each marker (Caspase‐3, BAX, BCL2, and PCNA). IntDen represents the sum of pixel values within the selected stained area and reflects both the extent and intensity of immunopositivity. For each tissue, at least five randomly selected fields per section were analyzed, and the mean IntDen values were calculated. These quantitative data were used for statistical comparison among groups.

### 2.9 Genetic Evaluation

The mitochondrial 16S rRNA region was selected as a mitochondrial molecular marker for sequence‐based comparison among the experimental groups. The obtained sequences were used to assess nucleotide variation and genetic distance among the groups. Genomic DNA was extracted from testes tissues of albino rats (*Rattus novergicus*), using DNA extraction method of QIAamp DNA Mini kit (Qiagen, Hidden, Germany) depending on the manufacturer's instructions. DNA was detected by agarose gel electrophoresis with a 100 bp DNA Ladder under UV light performed to amplify the mitochondrial 16S rRNA region using the forward primer 16Sar (5′‐CGCCTGTTTAACAAAAACAT‐3′) and the reverse primer 16Sbr (5′‐CCGGTCTGAACTCAGATCACGT‐3′), according to Simon et al. [31]. The PCR reactions were carried out using 10 pmol of each primer, approximately 100 ng of genomic DNA, and 12.5 µL of PCR master mix (OnePCR ready‐to‐use, Catalogue Number MB203‐0100, GeneDireX, Miaoli County, Taiwan) in a final reaction volume of 25 µL. PCR amplification was performed with an initial denaturation at 95°C for 2 min, followed by 35 cycles of denaturation at 95°C for 30 s, annealing at 50°C for 30 s, and extension at 72°C for 10 min. The PCR products were separated by electrophoresis on a 1.3% agarose gel stained with ethidium bromide. Sequencing of DNA done by Macrogen (South Korea), sequenced region of 16S rRNA in four groups was submitted in (GenBank/NCBI).

## 3 16S rRNA Genetic Analysis

Mitochondrial 16S rRNA was selected as a molecular marker to investigate sequence‐level genetic alterations in Rattus norvegicus following chronic ivermectin exposure and to evaluate the potential protective effects of folic acid and vitamin B12. The mitochondrial 16S rRNA region was selected because mitochondrial DNA is susceptible to oxidative stress‐associated damage and the 16S rRNA gene provides conserved regions suitable for reliable amplification together with variable sites that can be used to detect nucleotide sequence variations. Following amplification and sequencing, the obtained sequences were analyzed and compared with reference *Rattus norvegicus* sequences deposited in the NCBI GenBank database. Sequence similarities and nucleotide variations were assessed to characterize the genetic changes observed among the experimental groups

### 3.1 Statistical Analysis

The variability of the results was expressed as the mean ± standard deviation (SD). The data were statistically analyzed using one‐way analysis of variance (ANOVA) with GraphPad Prism software, followed by the Newman–Keuls post hoc test for multiple comparisons. The least significant difference (LSD) test was used to assess differences between treatments. Results were considered statistically significant when *p* < 0.05.

## 4 Results

### 4.1 Serum Testosterone and Sperms Count, Viability, and Motility

Table 1 shows the effect of treatment with IVM alone or in combination with FA and/or Vit.B12 for 30 days on the serum testosterone levels and semen profile in male albino rats. Results showed that IVM lowered testosterone (nmol/L) significantly (0.89 ± 0.07) compared to the control group (1.002 ± 0.24). Administration of FA and/or Vit.B12 in combination with IVM increased testosterone levels (0.97 ± 0.69 and 1.057 ± 0.12, respectively) compared to group received IVM alone and almost reached to control group level.

**TABLE 1:** The effect of treatment with Ivermectin alone or in combination with Folic Acid and/or Vitamin B12 on serum testosterone levels and semen profile in male albino rats.

Parameters	Testosterone (nmol/L)	Sperms count	Died sperms %	Live sperms %
Ctrl group	1.002 ± 0.24 ^a^	147.0 ± 43.84 ^a^	5.50 ± 0.70 ^b^	94.50 ± 0.70 ^a^
IVM group	0.892 ± 0.07 ^b^	84.33 ± 6.65 ^b^	44.33 ± 6.02 ^a^	55.67 ± 6.02 ^b^
IVM+FA group	0.970 ± 0.60 ^a^	157.7 ± 47.08 ^a^	5.00 ± 3.60 ^b^	95.00 ± 5.00 ^a^
IVM+Vit.B12 group	1.057 ± 0.12 ^a^	176.5 ± 68.59 ^a^	12.50 ± 1.75 ^b^	87.50 ± 3.53 ^a^

Values are means ± S.D. Values with different superscript (a, b) in the same column are significantly different at *p* < 0.05.

Ctrl group: Control group; IVM group: Ivermectin group; IVM + FA group: Ivermectin + Folic acid group; IVM + Vit. B12 group: Ivermectin + Vitamin B12 group.

Results revealed that IVM led to significant decreasing in sperm count and live sperms and increasing died sperms compared to the control group. The groups received FA and/ or Vit B12 in combination with IVM have good results where a significant rise of sperm count, percentage of live sperms and significant lowering on the percentage of died sperms were observed compared to the group received IVM alone and changes of the values of these groups made it almost resemble control group.

Also, the group received Vit.B12 increased sperm count with nonsignificant change compared to the control and the group received IVM, increased live sperms and decreased died sperms with significant change compared to the group received IVM. According to the control group, this group showed significant decrease in live sperms and nonsignificant increase in died sperms.

As shown in Table 2, the group of rats received IVM had nonprogressive motility compared to the control group. In contrast, group of rats received FA in combination with IVM had a satisfied progressive motility almost similar to the control group with nonsignificant difference found between these groups. The group received Vit.B12 with IVM had a significant increase in the progressive motility of the sperm and a significant decrease in nonprogressive motility compared to group received IVM alone and control group also.

**TABLE 2:** The effect of treatment with Ivermectin alone or in combination with Folic Acid and/or Vitamin B12 on sperm motility in male albino rats.

Parameters	Progressive motility	Non‐progressive motility
Ctrl group	35.00 ± 7.07^b^	65.00 ± 7.07 ^b^
IVM group	000.00000^c^	100^a^
IVM + FA group	31.00 ± 5.47^b^	69.00 ± 5.47^b^
IVM + Vit.B12 group	66.50 ± 4.95^a^	33.50 ± 4.95^c^

Values are means ± S.D. Values with different superscript (a, b, and c) in the same column are significantly different at *p* < 0.05.

Abbreviations: Ctrl group, control group; IVM group, ivermectin group; IVM + FA group, ivermectin + folic acid group; IVM + Vit. B12 group, ivermectin + vitamin B12 group.

### 4.2 Assessment of Oxidative Stress and Antioxidant Profile

Table 3 shows oxidative stress and the antioxidant profile in the control group compared to other groups treated with IVM alone or in combination with folic Acid and/or Vitamin B12 in adult male albino rats. The level of MDA in the tissue of the testis was significantly increased in IVM group compared to the other tested groups. Slight rise in the levels of MDA was observed in the IVM + FA group and IVM + Vit.B12 group, however this rise was nonsignificant. The levels of SOD and CAT in the tissue of the testis were significantly decreased in IVM group compared to the other tested groups, while the IVM + FA group and IVM + Vit.B12 group showed a significant increase in the levels of the same previously mentioned parameters. Nonsignificant decrease was observed in the levels of TAC in the IVM group compared to the Ctrl group; whereas both groups of the IVM + FA and IVM + Vit.B12 showed significant increase compared to the ctrl and IVM groups.

**TABLE 3:** Oxidative stress and the antioxidant profile in the control group compared to other groups treated with Ivermectin alone or in combination with Folic Acid and/or Vitamin B12 in adult male albino rats.

Parameters	MDA (nmol/ml)	SOD (U/ml)	CAT(U/ml)	TAC(U/ml)
Ctrl group	7.57 ± 0.50^b^	43.28 ± 2.56^b^	129.4 ± 1.25^a^	34.35 ± 1.15^b^
IVM group	18.23 ± 1.03^a^	31.05 ± 3.06^c^	70.13 ± 1.40^b^	31.66 ± 1.22^b^
IVM+FA group	10.62 ± 0.69^b^	98.65 ± 7.52^a^	103.3 ± 5.82^a^	61.17 ± 2.13^a^
IVM+Vit.B12 group	9.65 ± 0.48^b^	85.23 ± 6.97^a^	95.95 ± 4.18^a^	48.72 ± 3.02^a^

Values are means ± S.D. Values with different superscript (a, b, and c) in the same column are significantly different at *p* < 0.05.

Abbreviations: Ctrl group, control group; IVM group, ivermectin group; IVM + FA group, ivermectin + folic acid group; IVM + Vit. B12 group, ivermectin + vitamin B12 group; MDA, malondialdehyde; SOD, superoxide dismutase; CAT, catalase; TAC, total antioxidant capacity.

### 4.3 Histological Findings of the Testis

Control testicular sections showed normal seminiferous tubules with regular outlines containing complete spermatogenic series. Spermatogonia with dark nuclei were located near the basement membrane, while primary and secondary spermatocytes occupied the middle layers. Early and late spermatids appeared near the lumen, which contained numerous mature spermatozoa. Sertoli cells appeared tall and pyramidal with pale nuclei and prominent nucleoli, and Leydig cells were seen in the interstitial spaces with acidophilic cytoplasm and vesicular nuclei (Figure 1). In contrast, the IVM‐treated group exhibited disrupted tubular architecture, irregular tubules, degeneration of germinal epithelium, infiltration, and vacuolation, with lumina mostly devoid of spermatozoa, indicating impaired spermatogenesis. Meanwhile, both IVM + FA and IVM + Vit. B12 groups showed marked structural improvement, with seminiferous tubules closely resembling the control group, suggesting a protective effect against IVM‐induced testicular damage.

**FIGURE 1:**
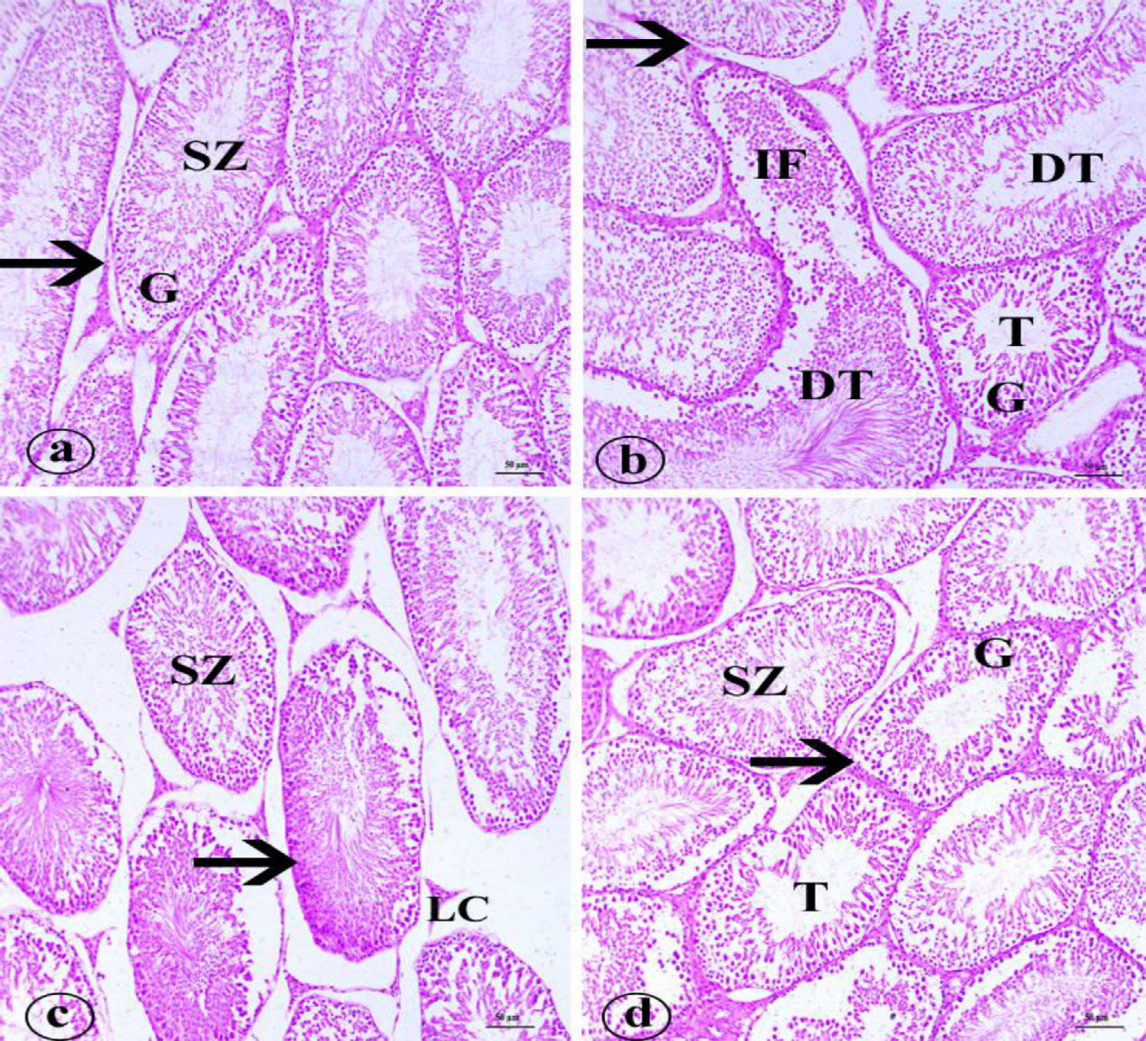
(a–d) Photomicrograph of H&E‐stained rat testis of different experimental groups. (a) The control group shows normal histological structure, seminiferous tubules (black arrows), lined by a series of spermatogenic cells (G) and mature sperms are seen in the lumen (SZ). The seminiferous tubules are separated by connective tissue which contains Leydig cells (LC). (b) The IVM group shows different pathological lesions, such as disrupted tubule architecture (DT), slight degeneration, inflammatory cells infiltration (IF), and few tubules reveal degenerated germinal epithelium with absence of mature spermatozoa in the lumen (T). (c) IVM + FA group (d) and IVM + Vit.B12 group—preserves their control character to a certain degree with slight degeneration and few tubules reveal degenerated germinal epithelium with absence of mature spermatozoa in the lumen. (H & E Scale bar = 50 µm).

### 4.4 Immunohistochemical Reactions of the Testis

Immunohistochemistry for cleaved caspase‐3 (Figure 2), an apoptosis effector, revealed that IVM substantially enhanced apoptotic activity in testis compared to control rat (Figure 2a). Conversely, cleaved caspase‐3 immunoexpression was downregulated by both IVM+ FA group (Figure 2c), and IVM+ Vit.B12 (Figure 2d) compared to the IVM group (Figure 2b). Thus IVM+ Vit.B12 was more effective than IVM + FA at inhibiting IVM‐induced apoptosis in rat testis.

**FIGURE 2:**
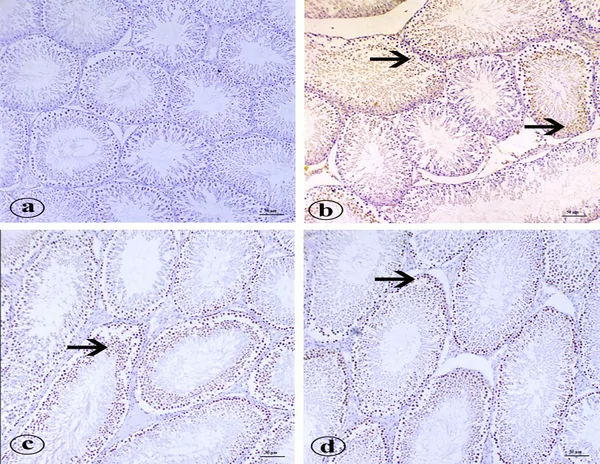
(a–d) Immunohistochemical staining for cleaved caspase‐3 protein expression levels in testis tissue of male albino rat from (a) control group (negative reaction), (b) IVM group (strong positive reactions), (c) IVM + FA group, and (d) IVM + Vit.B12 group (mild to moderate reactions) arrows indicating positive reactions. Scale bar = 50 µm.

The quantitative analysis (Figure 3) revealed a significant increase in the immunohistochemical expression of Caspase‐3 in the IVM‐treated group compared to the control group, indicating enhanced apoptotic activity. However, coadministration of folic acid or vitamin B12 with IVM markedly reduced Caspase‐3 immunoreactivity when compared to the IVM group, approaching near‐normal levels. These findings suggest that both folic acid and vitamin B12 possess potential protective effects against ivermectin‐induced apoptotic changes in tissue.

**FIGURE 3:**
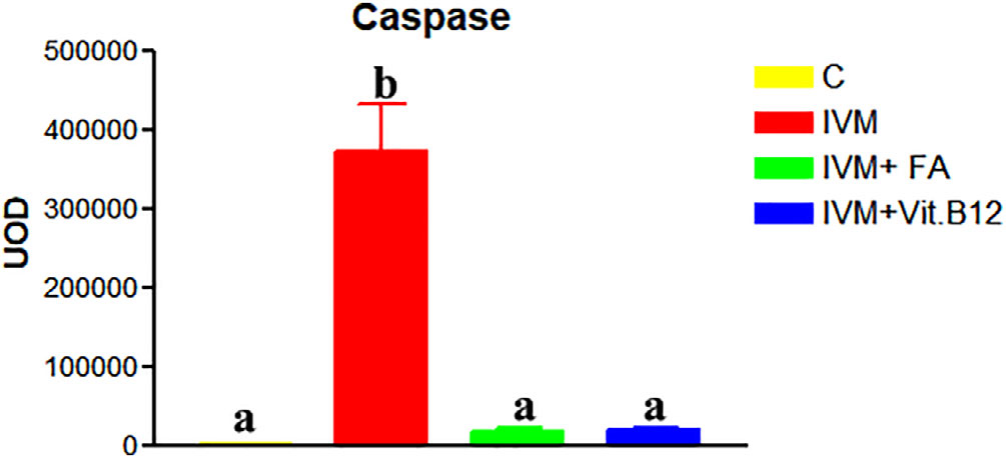
Quantitative analysis of the immunohistochemical expression of Caspase‐3 in the Control, IVM, IVM + FA, and IVM + Vit. B12 groups. Data are expressed as mean ± standard error of the mean (SEM) of unit optical density (UOD) values measured using ImageJ software. Statistical analysis was performed using one‐way ANOVA followed by Newman–Keuls post hoc test. A *p* < 0.05 was considered statistically significant.

Immunohistochemistry for the apoptosis marker Bax (Figure 4) revealed that IVM substantially enhanced apoptotic activity in the testis (Figure 4b) compared to control rat (Figure 4a). Conversely, Bax immunoexpression was downregulated in both IVM + FA group (Figure 4c), and IVM + Vit.B12 (Figure 4d) group compared to the IVM group (Figure 4b). Thus IVM + Vit.B12 was more effective than IVM + FA in inhibiting IVM‐induced apoptosis in rat testis.

**FIGURE 4:**
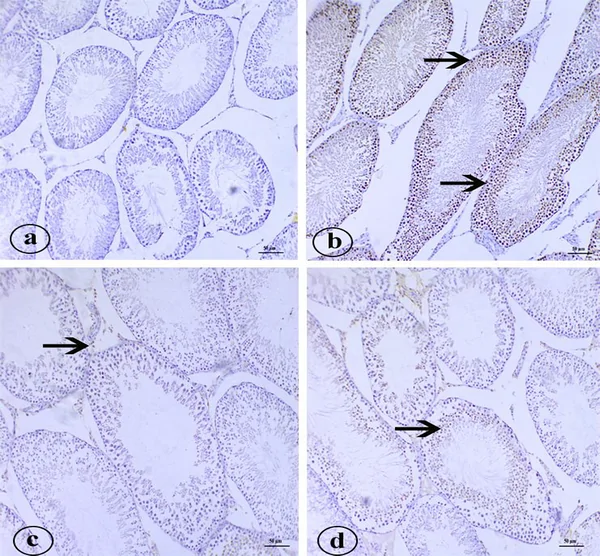
(a–d) Immunohistochemical staining for Bax expression levels in testis tissue of male albino rat from (a) control group (negative reaction), (b) IVM group (strong positive reactions), (c) IVM + FA group, and (d) IVM + Vit.B12 group (mild to moderate reactions) arrows indicating positive reactions. Scale bar = 50 µm.

The quantitative analysis revealed a significant increase in the immunohistochemical expression of BAX (Figure 5) in the IVM‐treated group compared to the control group, indicating enhanced apoptotic activity. However, coadministration of folic acid or vitamin B12 with IVM in the IVM + FA and IVM + Vit.B12 groups markedly reduced BAX immunoreactivity when compared to the IVM group, approaching near‐normal levels. These findings suggest that both folic acid and vitamin B12 possess potential protective effects against ivermectin‐induced apoptotic changes in tissue.

**FIGURE 5:**
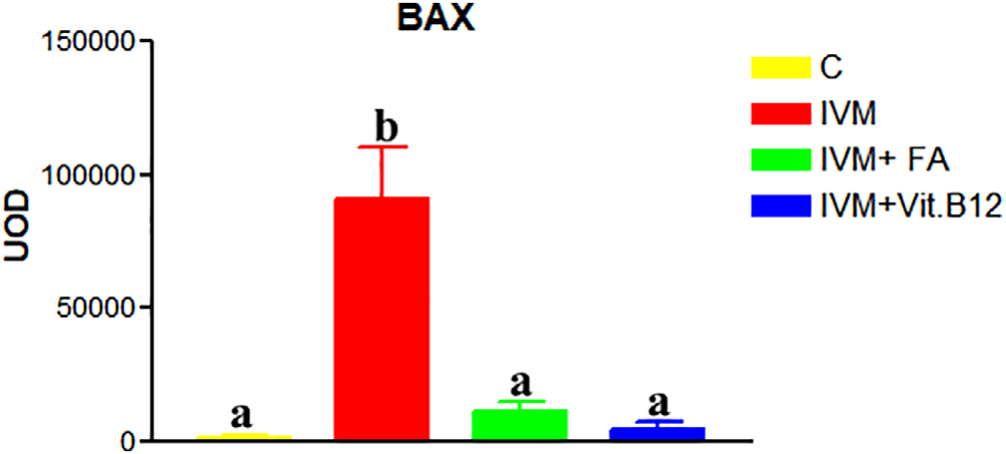
Quantitative analysis of the immunohistochemical expression of BAX in the Control, IVM, IVM + FA, and IVM + Vit.B12 groups. Data are expressed as mean ± standard error of the mean (SEM) of unit optical density (UOD) values measured using ImageJ software. Statistical analysis was performed using one‐way ANOVA followed by Newman–Keuls post hoc test. A *p* < 0.05 was considered statistically significant.

Immunohistochemistry for the apoptosis marker BCL2 (Figure 6) revealed that IVM substantially decreased in testis compared to control rat (Figure 6a). Conversely, BCL2 immunoexpression was upregulated by both IVM + FA group (Figure 6c) and IVM + Vit.B12 group (Figure 6d) compared to the IVM group (Figure 6b). Thus IVM + Vit.B12 was more effective than IVM+ FA in upregulating BCL2 levels in rat testis. For the quantitative analysis of the immunoreactivity, a marked reduction in BCL2 immunoreactivity (Figure 7) was observed in the IVM‐treated group compared to the control group, indicating suppression of anti‐apoptotic activity. Conversely, rats co‐treated with folic acid (IVM + FA) or vitamin B12 (IVM + Vit.B12) showed a significant increase in BCL2 expression compared with the IVM group.

**FIGURE 6:**
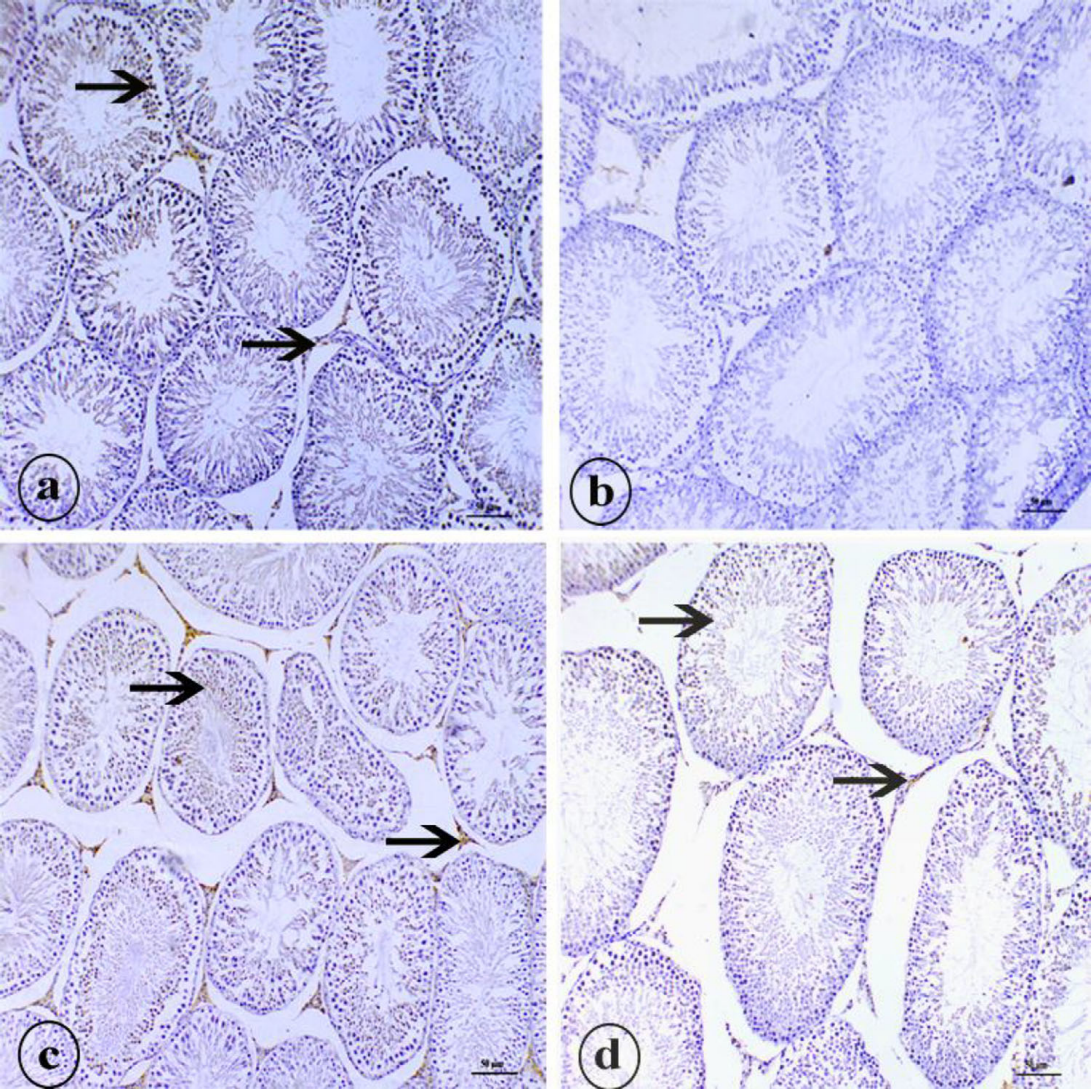
(a‐d) Immunohistochemical staining for BCL2 expression levels in testis tissue of male albino rat from (a) control group (positive reaction), (b) IVM group (negative reactions), (c) IVM + FA group, and (d) IVM + Vit.B12 group (positive reactions) and arrows indication positive reactions. Scale bar = 50 µm.

**FIGURE 7:**
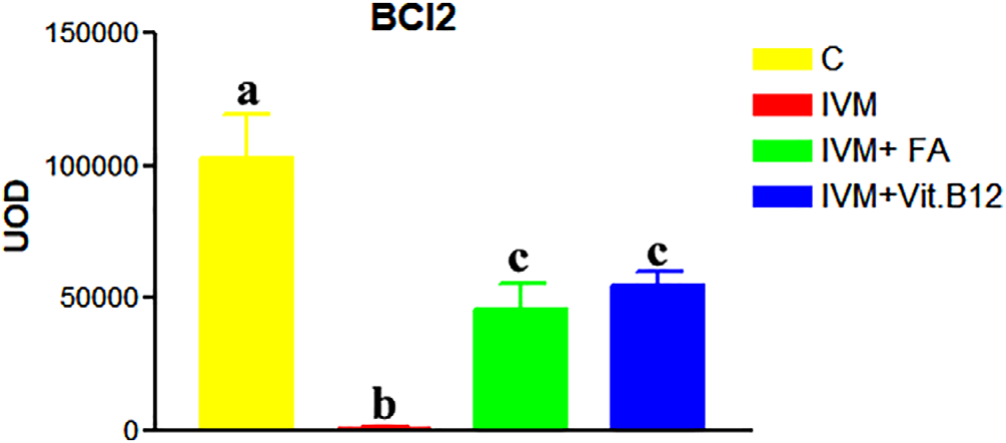
Quantitative analysis of the immunohistochemical expression of BCL2 in the Control, IVM, IVM + FA, and IVM + Vit.B12 groups. Data are expressed as mean ± standard error of the mean (SEM) of unit optical density (UOD) values measured using ImageJ software. Statistical analysis was performed using one‐way ANOVA followed by Newman–Keuls post hoc test. A *p* < 0.05 was considered statistically significant.

Immunohistochemical analysis of PCNA (Figure 8), a marker of cell proliferation, revealed that IVM substantially decreased in testis compared to control rat (Figure 8a). Conversely, PCNA immunoexpression was upregulated by both IVM + FA group (Figure 8c), and IVM + Vit.B12 group (Figure 8d) compared to the IVM group (Figure 8b). Thus IVM + Vit.B12 was more effective than IVM + FA in upregulating PCNA levels in rat testis. The immunohistochemical quantitative analysis demonstrated a significant decline in PCNA expression (Figure 9) in the IVM‐treated group relative to the control group, indicating reduced cellular proliferation in testicular tissue. In contrast, groups of IVM + FA and IVM + Vit.B12 exhibited a noticeable restoration of PCNA immunoreactivity, approaching control levels.

**FIGURE 8:**
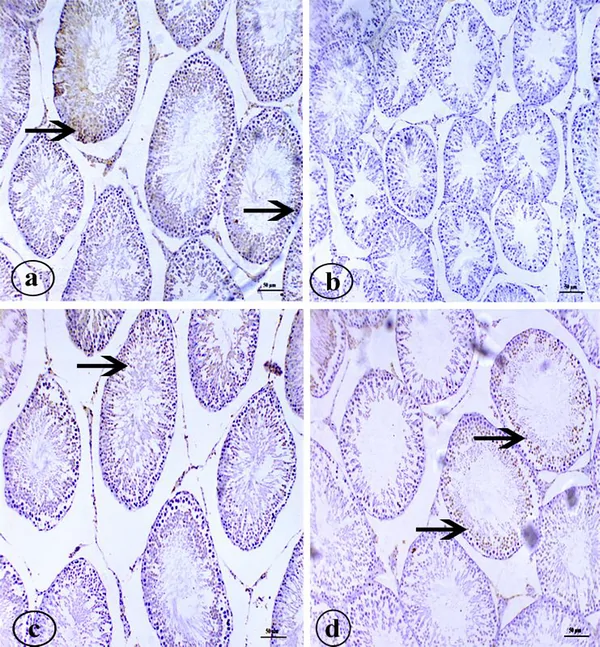
(a–d) Immunohistochemical staining for PCNA expression levels in testes tissue of male albino rat from (a) control group (positive reaction), (b) IVM group (negative reactions), (c) IVM + FA group and (d) IVM + Vit.B12 group showing positive reactions(arrows). Scale bar = 50 µm.

**FIGURE 9:**
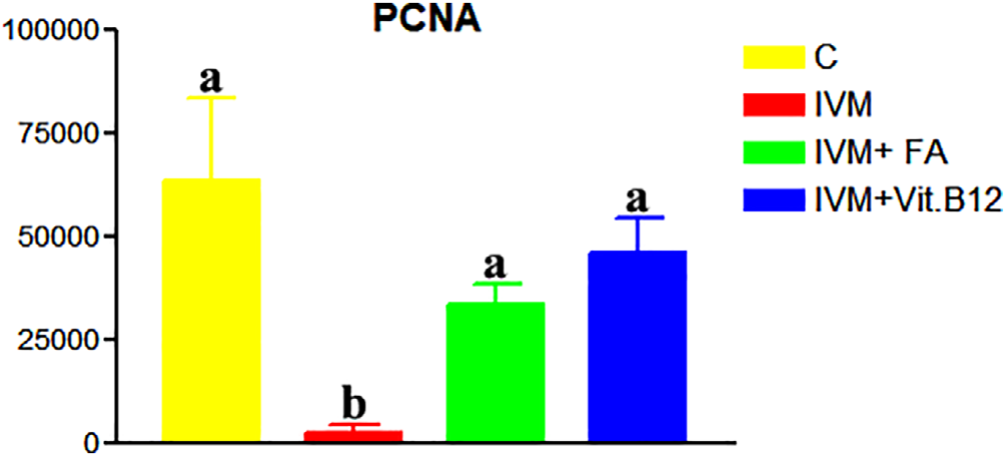
Quantitative analysis of the immunohistochemical expression of PCNA in the Control, IVM, IVM + FA, and IVM + Vit.B12 groups. Data are expressed as mean ± standard error of the mean (SEM) of unit optical density (UOD) values measured using ImageJ software. Statistical analysis was performed using one‐way ANOVA followed by Newman–Keuls post hoc test. A *p* < 0.05 was considered statistically significant.

### 4.5 Genetic Results

#### 4.5.1 PCR Amplification and Sequencing

PCR amplification and agarose gel electrophoresis resulted in a single band of 556 bp (base pair), sequenced region of 16S rRNA in four groups was submitted in (GenBank/NCBI) under accession numbers (PP320440.1, PP320441.1, PP320442.1, and PP320443.1) sequence was subjected to BLAST/N at National Centre for Biotechnology Information (NCBI).

Table 4 shows the nucleotide frequencies of 16S rRNA sequence in the all treated groups of tested rats of Rattus norvegicus (Rat species). Generally, A + T content is higher than C + G in all groups. The group received FA with IVM was the highest A + T content compared to all other groups while the group received Vit.B12 with IVM showed the lowest A + T content.

**TABLE 4:** Nucleotide frequencies of 16S rRNA sequence in all treated rats of *Rattus norvegicus* (Rat species).

Parameters	A%	T%	C%	G%	A + T	C + G
Ctrl group	33.40	27.46	19.48	19.67	60.68	39.15
IVM group	33.46	27.14	19.70	19.70	60.6	39.4
IVM + FA group	33.58	27.43	19.40	19.59	61.01	38.99
IVM + Vit.B12 group	33.21	27.26	19.68	19.86	60.47	39.54

*Note*: At the other side, the group received FA with IVM had the lowest C+G content whereas the group received Vit.B12 with IVM had the highest C+G content compared to other groups

Abbreviations: Ctrl group, control group; IVM group, ivermectin group; IVM + FA group, ivermectin + folic acid group; IVM + Vit. B12 group, ivermectin + vitamin B12 group.

Table 5 illustrates the genetic distance between all treated groups where the genetic distance between the control group and the group received IVM is 0.0018. The genetic distance between the group received IVM and the group of IVM+ FA is 0.0000 the same in genetic distance between IVM group and IVM + Vit.B12 group. Sequence analysis of the mitochondrial 16S rRNA region revealed nucleotide differences among the experimental groups, and pairwise genetic distance analysis was subsequently performed to evaluate the degree of sequence divergence among the groups.

**TABLE 5:** Pairwise genetic distances among the experimental groups based on mitochondrial *16S rRNA* sequence using Kimura 2‐ parameter model.

Parameters	Ctrl group	IVM group	IVM + FA group	IVM +Vit.B12 group
Ctrl group		0.0018	0.0000	0.0000
IVM group	0.0019		0.0018	0.0018
IVM + FA group	0.0000	0.0019		0.0000
IVM+Vit.B12 group	0.0000	0.0019	0.0000	

*Note*: Values represent the number of base substitutions per site between sequences. Analyses were conducted using the Kimura 2‐parameter model. A total of 556 nucleotide positions were included in the final dataset.

Abbreviations: Ctrl group, control group; IVM group, ivermectin group; IVM + FA group: ivermectin + folic acid group; IVM + Vit. B12 group, ivermectin + vitamin B12 group.

## 5 Discussion

Several studies have investigated the toxicity resulting from the excessive use of drugs intended for disease treatment [32, 33, 34] or those used as a protection against parasitic infestations [16]. Ivermectin is an antiparasitic agent used to treat a variety of infections, both in people and animals. Thus, its wide usage led to focus of the researchers to investigate its toxic effects following prolonged exposure with high dose. Recent research stated that IVM prolonged exposure with high dose induced toxic effect on the liver and kidney [35]. The current study examines the harmful effects of high doses of Ivermectin on reproductive health and testicular tissue, as well as the protective agents that may mitigate these effects. The ivermectin dose used in the present study (5 mg/kg/day, subcutaneously) represents a repeated high‐dose exposure and was selected based on a previously published experimental rat model [21]. Therefore, the present experimental design should be interpreted as a high‐dose toxicological model rather than a direct simulation of routine therapeutic exposure in humans. Accordingly, the severity of the reproductive and testicular effects observed in the present study should not be directly extrapolated to conventional clinical ivermectin exposure. Further studies using clinically relevant exposure levels are warranted to better define the translational significance of these findings.

Testosterone, a key sex hormone, has been investigated in several studies as an indicator of reproductive status [36, 37]. Testosterone levels were significantly affected by ivermectin (IVM) administration, which notably reduced its levels. This finding aligns with the study by El‐Maddawy and Abd El Naby [38] who suggested that IVM's impact on GABAergic receptors in the testes‐known for regulating testosterone production‐may be the underlying mechanism [9]. Conversely, folic acid (FA) administration led to an increase in testosterone levels, consistent with the findings of Fadl et al. [39], who reported that FA enhances blood supply to testicular tissues. Similarly, Gules et al. [40] demonstrated that FA mitigates bisphenol‐induced testicular toxicity. Moreover, Vitamin B12 (Vit.B12) also contributed to elevated testosterone levels in this study, supporting prior research by Rastegar Panah et al. [41], who emphasized Vit. B12's role in sperm health and testosterone production. Additionally, Ray et al. [42] found that Vit.B12 reversed the reduction in testosterone levels caused by sodium fluoride.

The assessment of semen picture in the current trial showed that IVM induced reduction in sperm count and motility. Similar results were announced by El‐Shobokshy et al. [43] and Sakthikarthikeyan et al. [44]. The decline in sperm motility was likely due to reactive oxygen species (ROS) damaging sperm DNA and proteins [7, 45], potentially inducing apoptosis in the cells of the seminiferous tubules responsible for spermatogenesis [11].

In contrast to the adverse effects of Ivermectin (IVM), folic acid (FA) administration improved sperm count and motility, aligning with the findings of Toghan et al. [16], who reported that rats received FA in combination with acetamiprid exhibited improved sperm count and motility compared to those receiving acetamiprid alone. Similarly, Lambrot et al. [46] and Ibrahim et al. [15] reported comparable results. This improvement is likely attributed to FA's antioxidant properties and its role in DNA synthesis, which is essential for spermatogenesis. Likewise, Vitamin B12 (Vit.B12) also enhanced sperm count and motility, supporting the results announced by Banihani [18], who demonstrated Vit.B12's role in improving sperm parameters in bovine semen.

Assessment of the oxidant/antioxidant profile is one of the most important aspects to be evaluated in males and females for assessing overall health status, and it has been investigated in several studies [47, 48, 49]. The assessment of the oxidant/antioxidant profile in the present study revealed that Ivermectin (IVM) induces oxidative damage, as MDA levels were significantly increased in the IVM‐treated group. Malonaldehyde (MDA), a biomarker of lipid peroxidation, has been widely used in previous researches, particularly in studies related to reproduction in both males and females [47, 50]. It has been detected in blood and tissues as an indicator of oxidative damage occurring within the body [48]. The increased oxidative stress due to IVM toxicity in this study is consistent with previous research that proved IVM induced oxidative stress and pathological alterations in the testes [38]. In contrast, FA and Vit.B12 in this study reduced MDA levels while increasing superoxide dismutase (SOD), catalase, and total antioxidant capacity (TAC) in the treated groups. The elevation of the antioxidant profile may explain the improvement in sperm health, as TAC has been linked to improved seminal parameters [51]. Additionally, their ability to decrease malondialdehyde (MDA)‐a compound responsible for cellular damage‐while increasing SOD and catalase plays a crucial role in cellular protection.

Histological findings of the testis assured the destroying effect of IVM, as the current study showed that control group has normal structure of the testis with regular rounded seminiferous tubules at different stages of spermatogenesis. In contrast, the testis manipulated with IVM showed irregular tubules and many of them lacking sperms which reflect impaired spermatogenic activity. Testis of both groups of IVM + FA and IVM + Vit.B12 showed a structure similar to the control group which indicate improving effect of these vitamins in maintaining healthy testis. Chronic exposure of the living body to bacterial and/or viral infection or parasitic infestation [36] or even to therapeutic drugs that leads to toxicity can result on destroying the tissue of the testis

Additionally, histochemical reactions in the testis support the results of reproductive hormone levels, oxidative stress assessments, and histological measurements. Caspase‐3 and Bax, which are the key markers of apoptosis, showed positive reactions in the IVM group, indicating increased apoptotic activity. PCNA, responsible for cell proliferation, and BCL2 showed negative reaction. Previous research has used caspase‐3 reaction as an indicator of apoptosis resulting from testicular damage and PCNA as an indicator of cell proliferation, demonstrating that a positive caspase‐3 reaction serves as a marker for tissue damage, while PCNA indicates tissue regeneration [52]. Similar findings were announced in previous researches that stated IVM treatment increased the expression of caspase‐3 in the seminiferous tubules of the testis [11, 43] and decreased the expression of the PCNA [43].

Caspase‐3 is one of the most important apoptotic markers investigated in several studies [53, 54]. In the current research, administration of FA and/or Vit. B12 with IVM inhibited Caspase‐3 and Bax activity and upregulated PCNA and BCL2, which reflect the role of these vitamins in remodeling the damage caused by IVM and keeping the cells of testis alive. Previous researches confirmed the protective effect of folic acid against toxicity by variant types of component. It was previously declared that folic acid when added to methomyl has the ability to downregulate the high expression of caspase‐3 in the testes [55]. Furthermore, folic acid elevated BCL2 expression either given alone or in combination with Atorvastatin [56]. Similarly, Vit. B12 downregulated the expression of caspase‐3 in the testes after exposure to cimetidine [57] and showed ability to upregulate PCNA expression that regenerate cells after testes exposure to methotrexate [58].

Mitochondrial 16S rRNA sequence analysis revealed a small genetic distance between the control and IVM groups (0.0019), indicating sequence variation between these groups. The genetic distances observed between the IVM and co‐treatment groups were similarly small, suggesting limited sequence divergence among the examined groups, which supports findings by Sweify et al. [59] who demonstrated IVM's genotoxicity. Similar genetic distance (0.0019) was found in the recent study that focused in assessment of the genotoxic effect of lead acetate in the testis [60]. Conversely, FA and Vit. B12 reduced the genetic variation caused by IVM, restoring the 16S rRNA gene to a state similar to that of the control group (no genetic distance, 0.0000). This observation is consistent with MacFarlane et al. [61], who highlighted FA's role in preventing genotoxicity. Furthermore, FA and Vit. B12 likely achieved this genetic protection through their involvement in DNA methylation, with FA providing methyl groups [62] and Vit. B12 supporting DNA synthesis and methylation [63, 64]. Notably, IVM‐induced oxidative stress [65], led to nucleic acid damage, including base adducts, which negatively influenced seminal parameters and testosterone levels. Mitochondrial 16S rRNA is a useful molecular marker for comparative sequence analysis because it contains conserved regions together with variable nucleotide positions. In Rattus norvegicus, comparative analysis of mitochondrial sequences has demonstrated nucleotide variation within the 16S rRNA region, supporting its use for detecting sequence‐level variation within this species [66]. In the present study, 16S rRNA sequencing was therefore used to assess nucleotide differences and genetic distances among the experimental groups. These findings provide complementary information on mitochondrial sequence variation among the groups and should not be interpreted as direct evidence of genomic DNA damage or genotoxicity.

## 6 Conclusion

Chronic IVM exposure adversely affected testosterone levels, sperm quality, testicular antioxidant status, histological architecture, and proliferative immunohistochemical markers in male rats. Coadministration of folic acid or vitamin B12 ameliorated these alterations. Mitochondrial 16S rRNA analysis revealed limited sequence variation among the experimental groups; however, the molecular findings should not be interpreted as direct evidence of genotoxicity. Further studies using dedicated DNA‐damage assays are warranted to clarify the potential genotoxic effects of chronic IVM exposure.

## Authors Contributions

Conceptualization: Y.A.A., Z.A.M., R.A.A. Data curation: Y.A.A., Z.A.M., S.A.M., A.M.M., R.A.A. Formal analysis: Y.A.A., Z.A.M., A.M.M., R.A.A. Investigation: Y.A.A., Z.A.M., S.A.M., A.M.M., R.A.A. Methodology: Y.A.A., Z.A.M., A.M.M., R.A.A. Writing – original draft: Y.A.A., Z.A.M., S.A.M., A.M.M., R.A.A. Writing – review and editing: Y.A.A., Z.A.M., A.M.M., R.A.A. All authors have read and approved the final manuscript.

## Funding

No fund was received from any agency.

## Ethics Statement

The experiment described in this thesis was performed in adherence to the zoology department, faculty of science. Guidelines on the care and use of laboratory animals and approval of the ethic committee of South Valley University were obtained before the study. All animal experiments complied with the ARRIVE guidelines and were carried out in accordance with the U.K. Animals (Scientific Procedures) Act, 1986 and associated guidelines, EU Directive 2010/63/EU for animal experiments.

## Consent

The authors have nothing to report.

## Conflicts of Interest

The authors declare no conflicts of interest.

## Data Availability

The data that support the findings of this study are available from the corresponding author upon reasonable request.
